# Efficacy of power training to improve physical function in individuals diagnosed with frailty and chronic disease: A meta‐analysis

**DOI:** 10.14814/phy2.15339

**Published:** 2022-06-06

**Authors:** Alexander B. Sklivas, Lauren E. Robinson, Timothy L. Uhl, Esther E. Dupont‐Versteegden, Kirby P. Mayer

**Affiliations:** ^1^ Department of Physical Therapy College of Health Sciences University of Kentucky Lexington Kentucky USA; ^2^ Center for Muscle Biology College of Health Sciences University of Kentucky Lexington Kentucky USA; ^3^ University of Kentucky Libraries Lexington Kentucky USA

**Keywords:** chronic disease, exercise, frailty, patient outcomes, physical function, power training

## Abstract

Muscle power training with emphasis on high‐velocity of concentric movement improves physical functionality in healthy older adults, and, maybe superior to traditional exercise programs. Power training may also be advantageous for patients with acute and chronic illnesses, as well as frail individuals. To determine the efficacy of power training compared with traditional resistance training on physical function outcomes in individuals diagnosed with frailty, acute illness or chronic disease. PubMed (MEDLINE), CINAHL, PEDro, Web of Science, and Google Scholar. (1) at least one study group receives muscle power training of randomized controlled trial (RCT) (2) study participants diagnosed as prefrail, frail or have an ongoing acute or chronic disease, condition or illness; (3) study participants over the age of 18; (4) publication in English language; (5) included physical function as the primary or secondary outcome measures. Two independent reviewers assessed articles for inclusion and graded the methodological quality using Cochrane Risk‐of‐Bias tool for RCTs. Fourteen RCTs met the inclusion criteria. In seven studies, muscle power training was more effective at improving physical function compared to control activities with a mean fixed effect size (ES) of 0.41 (*p* = 0.006; 95% CI 0.12 to 0.71). Power training and conventional resistance training had similar effectiveness in eight studies with a mean fixed ES of 0.10 (*p* = 0.061; 95% CI –0.01 to 0.40). Muscle power training is just as efficacious for improving physical function in individuals diagnosed with frailty and chronic disease when compared to traditional resistance training. The advantages of power training with reduced work per session may support power training as a preferential exercise modality for clinical populations. The findings should be interpreted with caution since generalizability is questioned due to the heterogeneity of patient populations enrolled and participants were relatively mobile at baseline.

## Graphical abstract

This systematic review assesses the efficacy of different training methods on chronically ill adults. The goal of this review was to determine if power training was more effective than traditional resistance training in chronically ill populations.

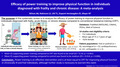

## 1 INTRODUCTION

Skeletal muscle dysfunction and weakness are common in patients with acute and chronic illnesses (Powers et al., 2016) and are typically the initial manifestation of frailty. Frailty is the clinically recognized state of vulnerability as a result of age‐associated decline (Fried et al., 2001; Walston et al., 2019; Xue, 2011). More recently frailty is recognized to be inter‐related to illness and disease, that is, frailty predicts negative consequences following illness (Augustin et al., 2016; Evered et al., 2020; Marengoni et al., 2021); acute illness (Bagshaw & Muscedere, 2017; De Biasio et al., 2020) or chronic disease may accelerate or exacerbate frailty (Chowdhury et al., 2017; Onder et al., 2018). Impairments in muscle health and function lead to deficits in functional mobility and ability to perform activities of daily living that negatively impact the quality of life (Reid & Fielding, 2012), which is observed in frailty and multiple clinical populations (Files et al., 2015; Johansen et al., 2003). An estimated 50% of patients surviving an intensive care unit admission will experience persistent skeletal muscle weakness (Puthucheary et al., 2013). Like‐wise, individuals with chronic obstructive pulmonary disease suffer skeletal muscle weakness and reduced exercise tolerance leading to limited functional mobility (Bernard et al., 1998). Exercise and physical activity can reverse or attenuate the loss of muscle function due to sarcopenia, cachexia, and ICU‐acquired weakness in clinical populations and during aging (Evans, 1996; Gould et al., 2013; Jones et al., 2015; Knols et al., 2005; Wischmeyer & San‐Millan, 2015). It is critically important to determine the exercise modality that induces the maximum benefit at the lowest frequency and intensity, since many clinical populations and the elderly have limited capacity for physical activity.

Muscular power, the ability of the muscle to generate work per unit of time, is a critical determinant of physical function (Bean et al., 2003, 2010; Reid & Fielding, 2012). Muscle power is fundamentally different from strength since it accounts for the velocity of movement (Winger et al., 2021). In aging, the rate of decline of muscle power is thought to occur earlier and twice as fast as the loss of muscle strength (Skelton et al., 1994). Moreover, deficits in muscle power have been suggested as a more important source of limiting functional mobility and activities of daily living in older adults, when compared to muscular strength losses (Bean et al., 2003; Izquierdo et al., 1999; Suzuki et al., 2001). Power training with high‐velocity of concentric movement has been compared to conventional strength or resistance training in a number of studies in community‐dwelling older men and women (Byrne et al., 2016; Henwood et al., 2008; McKinnon et al., 2017); these studies suggest that power training may be favorable to traditional training for improving functional performance (Bottaro et al., 2007; Byrne et al., 2016; Tschopp et al., 2011). Moreover, power training is believed to be less exhaustive often requiring “less total work performed per session,” therefore, potentially advantageous in clinical populations (Henwood et al., 2008; Sayers, 2007). However, the efficacy of power training, as well as conventional resistance training (CRT) has not been elucidated in clinical populations. Moreover, the feasibility and efficacy of power training in clinical populations compared to CRT has not been established. Power training is commonly performed in a supervised environment potentially preventing this training from being considered pragmatic (Byrne et al., 2016), but research studying the effects of power training in clinical populations is growing. Thus, the purpose of this systematic review is to analyze the efficacy of power training to improve physical function in individuals diagnosed with frailty, acute illness, or chronic disease compared to CRT.

## 2 MATERIALS AND METHODS

This systematic review is reported in accordance with the PRISMA statement for reporting systematic reviews and meta‐analyses of studies that evaluate healthcare interventions (Liberati et al., 2009). This protocol and the search strategies were registered in Prospero (ID 1335246).

### 2.1 Search strategy

The comprehensive search strategy was developed by a medical librarian (MR) in collaboration with the authors (KM, AS). Relevant studies were identified by searching MEDLINE via PubMed, CINAHL (EBSCOhost), Web of Science (Clarivate), and PEDro. We selected these databases based on institutional availability and discipline coverage. Additionally, a variety of Google Scholar searches were conducted and the first 10 results were exported from each search. Search strategies are provided in Table S1. The searches were conducted in December 2020 and the databases were searched from inception.

### 2.2 Study selection

Research studies were selected for inclusion if: (1) At least one study group received power training also referred to as high‐velocity training; (2) study participants were diagnosed as prefrail, frail, or have ongoing acute or chronic disease or illness; (3) study participants were over the age of 18; (4) publication in English language; (5) included physical function as the primary or secondary outcome measure. Frailty diagnosis was defined according to the original studies which included the frailty phenotype and the physical frailty approach (Robinson et al., 2015; Walston et al., 2019). Pre‐frail was defined as individuals are high risk of progressing to frailty meeting at least one or two criteria for frailty (Gill et al., 2006; Xue, 2011). Review articles, conference abstracts, and non‐peer‐reviewed articles were excluded. Secondary analyses of previously published research studies were excluded for final analysis.

### 2.3 Assessment of study quality

Quality assessment of all studies included in the final analysis was conducted by at least two independent reviewers (Kirby P. Mayer, Alexander B. Sklivas). Disagreements between initial reviewers were solved with discussion until consensus was achieved. The quality of randomized controlled trials was evaluated using the Cochrane risk of bias assessment (Higgins et al., 2011).

### 2.4 Study outcomes and definitions

The primary outcome of interest was the efficacy of power training, a form of resistance training, compared to conventional strength or CRT in improving physical function. Resistance training is defined as a form of periodic exercise whereby external stimuli provide progressive overload to skeletal muscles in order to make them stronger and often results in hypertrophy (Phillips & Winett, 2010). Power training was defined as a form of resistance exercise in which the concentric phase of the exercise is performed as fast as possible at a high‐velocity. CRT was defined as resistance training at low‐velocity or without the focus on velocity of movement. The efficacy of power training on improving physical function compared to a control group was a secondary outcome of interest. Control groups were considered in this analysis if participants were randomized to no intervention or light activity (i.e., walking program, yoga, or education) group, but did not receive power training or CRT as defined above. Efficacy was assessed based on improvement in physical function, defined as an objective measurement based on functional performance or functional capacity measured using validated outcome tests such as short physical performance battery (SPPB) or timed‐up and go (TUG) test.

### 2.5 Statistical analysis

Pooled descriptive statistics were calculated for age and sex. Mean and standard deviations were calculated from data as median and interquartile ranges using approach by Hozo et al. (2005). We computed Hedges adjusted g for individual effect size (ES) and variance of studies that assessed physical function using Comprehensive Meta‐Analysis Software (Biostat, Englewood, NJ) (Lipsey & Wilson, 2001), using 2 approaches: (1) Power training compared to CRT; (2) power training compared to control. We calculated fixed‐ and random‐effects models for these tests to ensure consistency in examining heterogeneity of the included studies. Effect sizes were categorized as small (<0.2), medium (0.2–0.8), and large (>0.8) according to Hedge’s g categories (Ottenbacher & Barrett, 1989). Heterogeneity statistics including Cochran’s Q and I‐squared values were calculated.

## 3 RESULTS

### 3.1 Study selection

**FIGURE 1:**
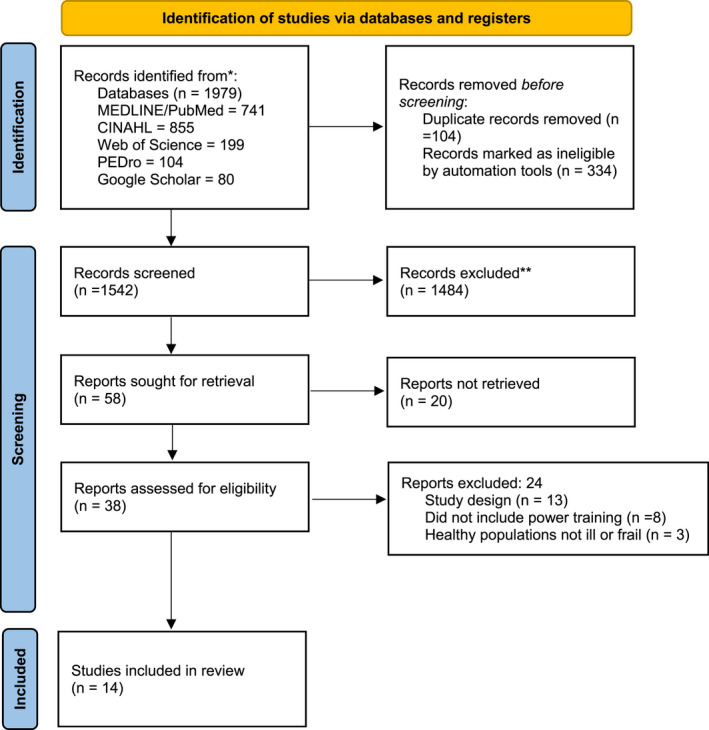
Prisma flow diagram.

Search of the online databases yielded 1548 titles, of which, all but 58 were excluded based on title, language, or full‐text not present. Of the 58 articles, 20 articles were excluded after abstract review. The remaining 38 studies were examined in full text. Of these studies, 24 were not included in the final review as study participants did not meet inclusion criteria (Figure 1). Two independent reviewers had three disagreements (95% agreement rate) during the abstract review which was solved with discussion. There were no disagreements during the full‐text review.

### 3.2 Patient population

A total of 711 patients were enrolled in the 14 studies with a pooled mean age of 71 ± 6.7 years of age and at least 62% female (2 studies not reporting sex). Study characteristics including demographics and intervention descriptions are summarized in Table 1. One trial enrolled older adults recovering from a total knee arthroplasty (Kelly et al., 2016). Five studies enrolled patients with neurological conditions including Parkinson’s Disease (PD) (Cherup et al., 2019; Ni et al., 2016; Paul et al., 2014), multiple sclerosis (MS) (Medina‐Perez et al., 2016), and cognitive impairment (Yoon et al., 2017). One trial studied power training in individuals classified as frail and five in those defined as pre‐frail (mobility‐limited older adults) (Bean et al., 2009; Cadore et al., 2014; Reid et al., 2008; Sayers et al., 2003; Webber & Porter, 2010; Zech et al., 2012). Studies on older adults with hyperglycemia (Jin et al., 2015) and adults with type II diabetes (Celes et al., 2017) were also included in the final analysis.

**TABLE 1:** Study characteristics

Author, Year	Participants	Inclusion Criteria (abbreviated)	Exclusion Criteria (abbreviated)	*n* (% female)	Age (SD)	Duration & frequency	Location & personnel
Cherup, 2019	Mild to moderate Parkinson’s disease	Adults diagnosed with PD (Hoehn and Yahr Stage I–III)	Participating in exercise; low score on Mini‐Mental State Examination; Recent MyoCardial Infarction	35 (34%)	71.1 (8.7)	12‐wks 2x week	University Kinesiology program; supervised
Celes, 2017	Adults with type II diabetes	Inactive patients with >10 years of Type 2 Diabetes	Enrolled in exercise program	30 (NR)	59.4 (15.6)	6‐wks 3x/wk	NR; “trained instructor”
Yoon, 2017	Older adults with mild cognitive impairment	> 65 years old, mild cognitive impairment, ability to walk 10m	Unstable cardiac disease, cerebrovascular disease, or musculoskeletal impairment,	58 (100%)	76 (3.8)	12‐wks 2x/wk	NR; Qualified instructor
Ni, 2016	Adults with PD	60–90 years old, idiopathic PD, able to ambulate 50 feet	Stage III PD, spinal fusion, orthopedic surgery, visual deficits, depression, dementia, greater than minimal assist for gait	41 (32%)	72.2 (7.0)	12‐wks 2x/wk	NR; Yoga instructor; trainer
Medina‐Perez, 2016	Adults with MS	Ages 18–65, MS diagnosis, EDS score between 3.0 and 6.0, ability to walk 20m, previously untrained	Conditions affecting muscle function or training protocol	77 (55%)	43.4 (9.5)	12‐wks 2x/wk	NR; Physical therapist
Kelly, 2016	Adults who had undergone primary unilateral total knee arthroplasty within the past 26 months	Ages 60–89, primary unilateral TKA, received inpatient and OPPT	Lower extremity or back pain independent of TKA, previous lower joint replacement, osteoporosis with a history of fracture, uncontrolled hypertension, diabetes, neurological disease, chest pain with stair climbing	38 (NR)	71.2 (6.8)	6‐wks 2x/wk	NR; Physical therapist
Jin, 2015	Elderly women with hyperglycemia	Hyperglycemia, fasting blood glucose >100 mg/dl	No medical disease or previous exercise habit	16 (100%)	75.2 (1.3)	12‐wks 3x/wk	NR
Paul, 2014	Adults with Parkinson Disease	>40, able to walk independently	Significant cognitive impairment, any unstable cardiovascular, orthopedic or neurological conditions	40 (33%)	66.3 (6.5)	12‐wks 2x/wk	University laboratory; Physiotherapist
Cadore, 2014	Older adults with frailty	>90–99 years old, frail	Absence of frailty, dementia, unable to walk indep, recent cardiac arrest, unstable medical condition	32 (70%)	91.9 (4.1)	12‐wks 4x/wk	Exercise gym; Physical Trainer
Zech, 2012	Older adults with pre‐frailty	Prefrail defined by Fried Fraility Scale, 65–94 years old	Depression, dementia, immunosuppressive drugs, COPD, IBS, angina pectoris, history of cancer, plasmacytoma	69 (71%)	77 (6.8)	12‐wks 2x/wk	Clinical setting; trained instructors
Webber, 2010	Older women with impaired mobility (pre‐frail)	Women >70 years old, mobility limitations	Unstable acute or chronic disease, participation in an exercise program in the last 6 months, neurological or musculoskeletal impairment interfering with the ability to participate	50 (100%)	77.0 (5.2)	12‐wks 2x/wk	NR
Bean, 2009	Older mobility limited adults (pre‐frail)	>65 years old, SPPB scores 4–10	Unstable acute or chronic disease, cognitive impairment, neuromusculoskeletal impairment limiting participation	138 (69%)	75.4 (6.8)	16‐wks 3x/wk	Outpatient rehab center
Reid, 2008	Older mobility limited adults (pre‐frail)	>65, community dwelling, mild‐moderate mobility impairments	Acute or terminal illness, myocardial infarction in the past 6 months, unstable cardiovascular disease, upper or lower extremity fracture in past 6 months or extremity amputation, hypertension, hormone therapy	57 (54%)	75.0 (7)	12‐wks 3x/wk	NR
Sayers, 2003	Older adults with self‐reported disability (pre‐frail)	>65 years old, community dwelling, walk independent, evidence of disability	Acute or terminal illness, recent myocardial infarction, unstable cardiovascular disease, recent fracture, cognitive impairment	30 (100%)	72.6 (2.1)	16‐wks 3x/wk	Human physiology laboratory; Exercise trainer

Note

Abbreviations: COPD, chronic obstructive pulmonary disease; IBS, irritable bowel syndrome; m, meter; MS, Multiple Sclerosis; PD, Parkinson Disease; NR, not reported; OA, osteoarthritis; SPPB, short physical performance battery; wk, week.

### 3.3 Study quality

Only one study was rated a potential for considerable risk of methodological bias and the remaining 13 studies were low to moderate risk (Supplemental Table 2).

### 3.4 Physical function

Studies assessed physical function using a diverse set of functional outcome tests (Table 2). The most common physical function performance‐based outcome measures were the SPPB and TUG utilized in eight studies, respectively.

**TABLE 2:** Study Outcomes and training description

Author, year	Study groups	Power training description	Results
Cherup, 2019	2 groups: Power training: strength training	Power training performed 10 exercises at 30–50% of 1RM with explosive motion at maximal velocity of the eccentric phases. Strength training performed same 10 exercises at 70% of 1 RM at controlled rate of movement (2–3 s)	Both power and strength training appear equally‐effective at improving measures of muscular strength and power; but neither group demonstrated improved functional performance.
Celes, 2017	2 groups: Low‐load high‐velocity resistance (power); recreational activities as control	Low‐load high‐velocity performing 5 exercises performed as fast as possible at moderate weight 60% 1RM, 3 sets of 8 repetitions	Significant improvement in rate of force development, sit‐to‐stand testing and 6MWT in power group compared to control, but TUG did not improve
Yoon, 2017	3 groups: High‐velocity power training; low‐speed strength training; control	Very low intensity elastic bands performing 40 minutes of exercises, 2–3 sets for 12–15 reps with power group performing as fast as possible	Power training was superior to resistance in higher changes in cognition, SPPB, TUG, grip strength and peak torque production
Ni, 2016	3 groups: Power training with pneumatic machines; yoga program with focus on movement speed; control	Power training: UE and LE exercises with pneumatic machines in a circuit, 3 sets, 12 reps at 50–75% of 1 RM. Yoga program was designed for movement speed	Both training groups produced significant improvement compared to control in BBS, TUG, and MiniBest‐Test; no differences between training groups.
Medina‐Perez, 2016	2 groups: High‐speed power training of knee extensors; control	Knee extension exercises on a weight stack machine twice per week, 3–4 sets of 4–10 reps at 40–70% MVIC as fast as possible	Power training significantly increased torque and MVIC compared control group
Kelly, 2016	2 groups: High‐velocity and low‐velocity training consisting of a multitude of functional movements	High‐speed curbs, stairs, and open‐chain resistive exercises	Functional performance significantly improved within each group from baseline, but was not different between the two training groups; only the high‐velocity group reported significant pain relief
Jin, 2015	2 groups: Muscle power training; control	High‐speed, low‐intensity whole body exercises were performed with elastic bands for 2 sets of 10 reps	Power group had significant improvements in blood glucose, adiponectin, interleukin, SPPB, and grip strength from baseline
Paul, 2014	2 groups: Leg muscle power training using pneumatic variable resistance equipment; low intensity control	3 sets of 8 reps as fast as possible targeting leg extensors, knee flexors, hip flexors, and hip abductors using pneumatic variable resistance equipment	Leg muscle power and strength was significantly improved in power group compared to the control; significant improvements in mobility and balance
Cadore, 2014	2 groups: Multi‐component exercise program with focus on high velocity; control	2 LE exercises and one UE exercise performed at 40%–60% 1RM for 8–10 reps combined with balance and gait training exercises	Significant improvements in gait velocity, TUG, 30s STS, balance, and incidence of falls
Zech, 2012	3 groups: Muscle power training, muscle strength training, control	2 sets of 15 reps o chest press, hip extension/flexion, hip abduction/adduction, calf raises, and chair rise as fast as possible	Both the power and strength training groups significantly improved SPPB; only the strength group experienced a decline in SPPB following detraining
Webber, 2010	3 groups: High‐velocity elastic bands; high‐velocity weights; control	Weights group performed 3 sets of 8–10 reps of ankle dorsiflexion and plantarflexion at 80% of 1RM as fast as possible; bands group performed 3 sets of 8 reps of dorsiflexion and plantarflexion as fast as possible	All groups demonstrated improvements in DF and PF, but only the power group with elastic bands demonstrated an improvement in movement time
Bean, 2009	2 groups: InVest training program with weight‐vest and high velocity of movement; traditional resistance strength training	Exercises addressing major UE and LE muscle groups as well as trunk while wearing a weight vest emphasizing a task‐specific movement as quickly as possible, 2 sets, 10 reps	Statistically power training with weight‐vest was superior to strength training at improvement muscle power, but not physical function measured by SPPB.
Reid, 2008	3 groups: High‐velocity power training; low‐velocity progressive resistance training; control	Power group performed 3 sets of 8 reps of leg press and knee extension as fast as possible at 70% of 1RM	Significant improvements were noted in power output and leg press specific power in the power group
Sayers, 2003	2 groups: Progressive resistance training; High‐velocity power training	High velocity of 3 sets, 8 reps using bilateral leg press machine and knee extensor pneumatic exercise equipment, as fast as possible 70% of 1RM	There was no difference in high‐velocity vs low‐velocity in functional performance or disability.

### 3.5 Performance‐based physical function ES

**FIGURE 2:**
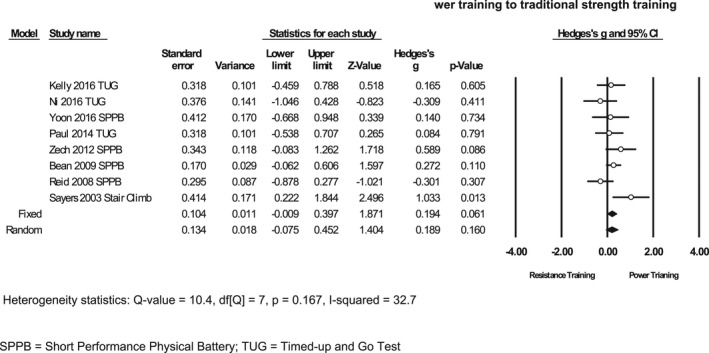
Effect size for performance‐based physical function comparing power training to traditional strength training.

Eight of the fourteen studies compared power training to CRT, which included populations of frailty and pre‐frailty, total knee arthroplasty, PD, and mild cognitive impairment. The mean ES for these studies was found to be small, demonstrating 0.19 in support of power training (SE 0.105; *p* = 0.061; 95% CI –0.01 to 0.40) (Figure 2). One additional study compared power training to CRT, but physical function data were reported aggregated, so could not be used in meta‐analysis. The authors provided an ES for combined groups reporting that power and resistance training did not significantly improve function (TUG).

**FIGURE 3:**
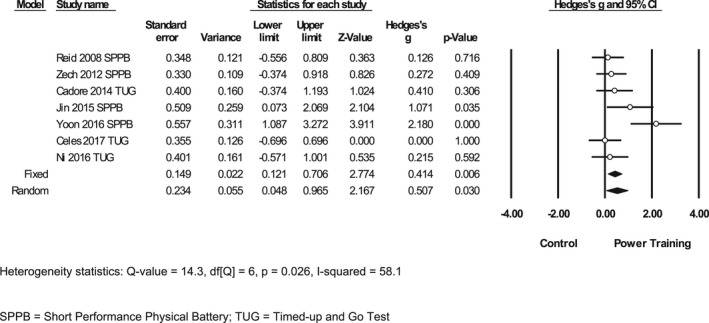
Effect size for performance‐based physical function comparing power training versus a control.

Seven studies compared the effectiveness of power training to improve physical function outcomes versus a control group (no intervention or light physical activity). The meta‐analysis of these studies resulted in a medium mean ES of 0.414 favoring power training (SE 0.149; *p* = 0.006; 95% CI 0.121–0.706, Figure 3). The seven studies included study populations such as older women with hyperglycemia, mild cognitive impairment, adults with type II diabetes, PD, frailty, and mobility limited older adults.

## 4 DISCUSSION

The findings of this systematic review support power training as an effective therapeutic intervention for improving physical function in adults diagnosed with frailty and patients with chronic medical conditions. Medium effect size indicates that power training is more effective than control conditions. Small effect sizes suggest that power training is not inferior to CRT, and may demonstrate potential for therapeutic benefit when implemented in specific patient populations. Small to medium effect sizes should be interpreted with caution due to heterogeneity in the included patient populations. Participants included in this meta‐analysis included individuals with pre‐existing orthopedic, neurologic, and metabolic conditions as well as a diagnosis of frailty and pre‐frailty. Thus, aggregated efficacy may not be representative of all clinical populations. The findings, however, provide preliminary evidence that muscle power training is efficacious for improving physical function in a variety of patient populations.

Physical function impairments commonly manifest from acute illness and chronic disease (Powers et al., 2016). Muscular weakness and dysfunction leading to deficits in functional mobility frequently hinder activities of daily living and negatively impact the quality of life for individuals with frailty, acute illness, and chronic disease (Alnahdi et al., 2012; LeBrasseur et al., 2006; Parry et al., 2015; Roshanravan et al., 2017). Deficits in muscular power may be targeted through power training to enhance muscle and physical function. Previously, studies have demonstrated that power training can improve physical function and maybe a superior training modality to traditional strength or resistance training in older adults (Bottaro et al., 2007; Henwood et al., 2008; Tschopp et al., 2011). Power training has been implemented in clinical populations and individuals with frailty. Frailty is defined as a clinical syndrome increases the risk of poor health outcomes such as falling, disability, hospitalization, and mortality (Cadore et al., 2014), and is associated with disability and comorbidity, but has distinct biologic bases that maybe independent of sarcopenia (Xue, 2011). In 2001, Fried and colleagues developed a standardized definition for frailty with established criteria, including skeletal muscle weakness (Fried et al., 2001). In this study, frailty was independently predictive of falls, worsening mobility, hospitalization, and mortality in older adults (Fried et al., 2001). Frailty is a clinical term that has been accepted across a wide range of conditions, diseases, and illnesses. An estimated 15%–45% of older adults admitted to nursing‐home are frail or pre‐frail and the prevalence of frailty increases steadily with chronic disease (Cesari et al., 2006; Fried et al., 2001). Moreover, the clinical diagnosis of frailty is common among younger critically ill patients, not just older adults (Cesari et al., 2006). Frail individuals have lower muscle density and muscle mass (Bagshaw et al., 2016). Frailty is driven by the loss of metabolically active cellular mass resulting from muscle loss and subsequently leads to reductions in resting metabolic and physical activity (Cesari et al., 2006). Therefore, there is a clinically meaningful, bidirectional relationship between frailty and acute illness (Bagshaw & Muscedere, 2017; De Biasio et al., 2020), as well as frailty and chronic diseases (Chowdhury et al., 2017; Onder et al., 2018). Moreover, the presence of acute illness (Files et al., 2015; Johansen et al., 2003) and chronic disease (Anagnostis et al., 2020; MacKinnon et al., 2018; Sepúlveda‐Loyola et al., 2020) increases the risk for muscle deficits and physical function impairments, independent of frailty. Individuals with frailty and chronic disease have high utilization of healthcare resources, loss of income, and progressive risk of mortality. Therefore, it is of critical concern to find therapeutic interventions that prevent, reverse, or mitigate deficits associated with frailty, disease and illness, and power training may be this therapy.

Power training has gained substantial traction as an exercise modality to improve physical function, especially in older adults and was recommended in a recent position statement (Fragala et al., 2019). Of clinical significance, muscle power is closely associated with mobility and physical function (Bean et al., 2003; Reid & Fielding, 2012). In addition, power training typically utilizes lighter weights or loads for exercises when compared to CRT thus enhancing the safety while still eliciting functional gains (Henwood et al., 2008). The optimal intensity, load, and repetitions for traditional resistance training remains unclear (Steib et al., 2010), likewise, the optimal dosage for power training has not been established. In the studies included in this review, power training was implemented with various modalities including pneumatic machines, elastic bands, free‐weights, and functional body movements at varied loads and repetitions. Thus, the optimal delivery of power training in clinical populations has not been defined. Despite the diverse approach to power training regimens, the findings of this systematic review support implementation for clinical populations. It should be noted that safety was not a focus of this analysis and should be considered before having individuals with disease and condition chronic engage in power training.

In this systematic review, we demonstrate that power training improves physical function when compared to control. A systematic review comparing the differences of power training versus CRT on muscle hypertrophy in older, though not necessarily diseased populations, found power training to be as effective as resistance training (Orssatto et al., 2020). Another systematic review comparing the effects of CRT versus power training on functional performance in older adults found power training to be as effective as CRT in improving functional performance in older adults (Tschopp et al., 2011), similar to our own results. A third systematic review demonstrated thigh velocity training may be superior to moderate velocity training; although the studies meeting eligibility only included adults ≥60 years of age, with many studies utilizing healthy adults (Rosa Orssatto et al., 2019). Our systematic review includes three overlapping studies (Bean et al., 2009; Yoon et al., 2017; Zech et al., 2012) all of which were classified as “pre‐frail” category. The stage of pre‐frailty may represent a transition from healthy older community‐dwelling adult to the individual at risk of negative health outcomes and thus minimal overlap is noted in these systematic reviews. Our review, however, incorporates a diverse array of clinical populations including individuals with neurologic, cardiovascular, and orthopedic conditions. The culmination of data, supports that randomized controlled trials with larger sample sizes are necessary to determine if power training is more efficacious than CRT for improving physical function. Moreover, trials in specific patient populations are necessary to improve generalizability and reproduce results found in this systematic review. Continued research is imperative as several limitations in the original studies were present including potential bias in methodology and small sample sizes, which is a similar theme noted in the previous systematic reviews (Orssatto et al., 2020; Rosa Orssatto et al., 2019; Tschopp et al., 2011).

Our systematic review is not without limitations. As mentioned, the inclusion of all clinical populations reduced generalizability. Secondly, findings are limited by heterogeneity in reporting of physical function outcomes and thus we aggregated SPPB, TUG, and chair rise test to calculate effect sizes. The physical function may encompass a wide variety of mobility or movement tasks to assess patient’s ability or capacity to perform activities of daily living. Secondary limitations include the potential for methodological bias as blinding of participants and participants is challenging when delivering a physical intervention. Finally, the baseline health of participants in the included studies should be considered when interpreting our findings. Interestingly, the study populations were considered chronically diseased or frail for inclusion, however, most participants had a high baseline functional and mobility status. Study enrollment criteria frequently excluded patients unable to ambulate. Hence, the interpretations of our data should only be applied to populations with chronic disease with mild functional impairments and not to those individuals with more significant physical disabilities. It should also be noted, that no studies included patients hospitalized or recently discharged for acute illness.

## 5 CONCLUSION

Power training is an effective intervention and is at least equal to CRT for improving physical function in chronically ill and frail individuals, although further study is necessary to warrant this claim. The findings are limited as the included studies only enrolled individuals with low acuity of disease, therefore, generalizability to populations with severe illness is questioned. Additional research is necessary to confirm the efficacy of power training in different patient populations such as those with critical illness, cancer, and chronic obstructive pulmonary disease. Future research should examine the appropriate dosing, frequency, intensity, and duration of power training to enhance safety and maximize potential benefit.

## CONFLICTS OF INTEREST

The authors have no conflicts of interest to disclose.

## ETHICS STATEMENT

This systematic review is reported in accordance with the PRISMA statement for reporting systematic reviews and meta‐analyses of studies that evaluate healthcare interventions. This protocol and the search strategies were registered a priori in Prospero (ID 1335246). The study precluded need for institutional review board approval.

## AUTHOR CONTRIBUTIONS

Alexander B. Sklivas and Kirby P. Mayer were involved in all stages of the study including concept, data collection and analyses, and dissemination. Lauren E. Robinson developed and performed the systematic searches and participate writing methods. Timothy L. Uhl and Esther E. Dupont‐Versteegden provided research oversight, assisted with interpretations of the data, and edited manuscript. All authors reviewed and approved the final version.

## Supporting information



Table S1‐S2
