# Supplementary material for: Efficacy of power training to improve physical function in individuals diagnosed with frailty and chronic disease: A meta‐analysis
Source: Physiol Rep. 2022 Jun 6;10(11):e15339. doi: 10.14814/phy2.15339 (PMC9170947; doi:10.14814/phy2.15339)
Supplement: Supplementary file 1 — Table S1‐S2 [file PHY2-10-e15339-s001.docx]

**Supplemental Table 1: Search Strategies**

| Search Queries | Database | Results |
| --- | --- | --- |
| ((((((((((((((((((((((((((((((((((((((((((((((((((((((((((((((((((((((((((("Frail elderly"[Mesh])) OR ("pre frail*"[tiab])) OR ("frail elderly*"[tiab])) OR (frail*[tiab])) OR ("older adult*"[tiab])) OR ("Stroke"[Mesh])) OR (stroke*[tiab])) OR ("cerebrovascular accident*"[tiab])) OR (CVA[tiab])) OR (CVAs[tiab])) OR ("Osteoarthritis"[Mesh])) OR ("Osteoarthriti*"[tiab])) OR ("Osteoarthro*"[tiab])) OR ("Degenerative Arthriti*"[tiab])) OR ("Arthrosis"[tiab])) OR ("Arthroses"[tiab])) OR ("osteoarthrosis deformans"[tiab])) OR ("Parkinson Disease"[Mesh])) OR ("Parkinson's Disease*"[tiab])) OR ("Parkinson Disease*"[tiab])) OR ("Primary Parkinsonism"[tiab])) OR ("Paralysis Agitans"[tiab])) OR ("Lewy Bodies"[Mesh])) OR ("lewy bod*"[tiab])) OR ("Lewy Neurites"[tiab])) OR ("Diabetes Mellitus, Type 2"[Mesh])) OR ("Type 2 Diabetes"[tiab])) OR ("Maturity Onset Diabetes*"[tiab])) OR ("Type 2 Diabetes Mellitus"[tiab])) OR (MODY[tiab])) OR ("Diabetes Mellitus*"[tiab])) OR (T2DM[Title/Abstract])) OR ("Chronic Disease"[Mesh])) OR ("Chronic Disease*"[tiab])) OR ("Chronic Illness*"[tiab])) OR ("chronically ill*"[tiab])) OR ("Multiple Sclerosis"[Mesh])) OR ("Multiple Sclerosis*"[tiab])) OR ("Disseminated sclerosis*"[tiab])) OR ("Heart Failure"[Mesh])) OR ("heart fail*"[tiab])) OR ("Cardiac Failure"[tiab])) OR ("Heart Decompensation"[tiab])) OR ("Myocardial Failure"[tiab])) OR ("Congestive Heart Failure"[tiab])) OR ("Cardiovascular Diseases"[Mesh])) OR ("Cardiovascular Disease*"[tiab])) OR ("Vascular Diseases"[Mesh])) OR ("Vascular Disease*"[tiab])) OR ("Arthritis, Rheumatoid"[Mesh])) OR ("Rheumatoid Arthritis*"[tiab])) OR (RA[tiab])) OR ("Autoimmune Diseases"[Mesh])) OR ("Autoimmune Disease*"[tiab])) OR ("Cystic Fibrosis"[Mesh])) OR ("Cystic Fibrosis*"[tiab])) OR (Mucoviscidosis[tiab])) OR ("Neuromuscular Diseases"[Mesh])) OR ("Neuromuscular Disease*"[tiab])) OR ("Movement Disorders"[Mesh])) OR ("Movement Disorder*"[tiab])) OR ("Dyskinesia Syndrome*"[tiab])) OR (("Walking"[Mesh] OR ambulat*[tiab] OR walk*[tiab] OR movement*[tiab]) AND difficulty[tiab])) OR ("Mobility Limitation"[Mesh])) OR ("Mobility Limitation*"[tiab])) OR ("Independent Living"[Mesh])) OR ("Independent Living"[tiab])) OR ("community dwelling"[tiab])) OR ("Disability Evaluation"[Mesh])) OR ("Disability Evaluation"[tiab])) OR ("Arthroplasty"[Mesh])) OR ("Arthroplasty"[tiab])) AND ((((((((((((((((((("Exercise Therapy"[Mesh]) OR ("exercise therap*"[tiab])) OR ("remedial exercis*"[tiab])) OR ("Resistance Training"[Mesh]))) OR ("rehabilitation exercis*"[Title/Abstract])) OR ("resistance training"[tiab])) OR ("strength training"[tiab])) OR (("weight") AND (lift*[tiab] OR bearing[tiab]) AND (strength*[tiab] OR exercis*[tiab] OR program*[tiab]))) OR ("Muscle Strength"[Mesh])) OR ("Muscles"[Mesh])) OR ("muscle strength*"[tiab])) OR ("Arthrogenic Muscle Inhibition*"[tiab])) OR ("Muscle Strength Dynamometer"[Mesh])) OR ("Muscle Strength Dynamometer*"[tiab] or "dynamometer*"[tiab])) OR ("Physical Fitness"[Mesh])) OR ("Physical Fitness*"[tiab])) OR ("Physical Endurance"[Mesh])) OR ("Physical Endurance*"[tiab]) OR ("multicomponent exercise*"[tiab]))) AND ((((((((((((((((((("muscle power*"[tiab]) OR ("muscle quality"[tiab])) OR ("power output*"[tiab])) OR ("high velocity*"[tiab] AND "low load*"[tiab])) OR ("LLHV"[tiab])) OR ("power training"[tiab])) OR ("functional performance"[tiab])) OR ("rate of force*"[tiab])) OR ("rate of velocity*"[tiab])) OR ("training load"[tiab])) OR (high[tiab] AND low[tiab] AND "external resistance"[tiab])) OR ("muscle volume*"[tiab])) OR ("force velocity*"[tiab])) OR ("Muscle Fatigue"[Mesh])) OR ("muscl*"[tiab] AND "fatigue"[tiab])) OR ("Muscle hypertrophy"[tiab])) OR ("Functional outcome*"[tiab])) OR ("Muscle mass"[tiab])) OR ("peak power"[tiab]))) | MEDLINE via PubMed | 741 |
| ( ( (MH "Frail Elderly") OR (MH "Stroke+") OR (MH "Osteoarthritis+") OR (MH "Arthritis, Rheumatoid+") OR (MH "Arthritis") OR (MH "Parkinson Disease") OR (MH "Parkinsonian Disorders+") OR (MH "Lewy Body Disease") OR (MH "Diabetes Mellitus") OR (MH "Diabetes Mellitus, Type 2") OR (MH "Chronic Disease+") OR (MH "Critical Illness") OR (MH "Multiple Sclerosis+") OR (MH "Heart Failure+") OR (MH "Cardiovascular Diseases+") OR (MH "Vascular Diseases+") OR (MH "Autoimmune Diseases+") OR (MH "Cystic Fibrosis") OR (MH "Neuromuscular Diseases+") OR (MH "Movement Disorders+") ) OR ( TI ( "pre frail*" OR "frail elderly*" OR frail* OR "older adult*" OR stroke* OR "cerebrovascular accident*" OR CVA OR CVAs OR Osteoarthriti* OR Osteoarthro* OR "Degenerative Arthriti*" OR Arthrosis OR "Arthroses" OR "osteoarthrosis deformans" OR "Parkinson's Disease*" OR "Parkinson Disease*" OR "Primary Parkinsonism" OR "Paralysis Agitans" OR "lewy bod*" OR "Lewy Neurites" OR "Type 2 Diabetes" OR "Maturity Onset Diabetes*" OR "Type 2 Diabetes Mellitus" OR MODY OR "Diabetes Mellitus*" OR T2DM OR "Chronic Disease*" OR "Chronic Illness*" OR "chronically ill*" OR "Multiple Sclerosis*" OR "Disseminated sclerosis*" OR "heart fail*" OR "Cardiac Failure" OR "Heart Decompensation" OR "Myocardial Failure" OR "Congestive Heart Failure" OR "Cardiovascular Disease*" OR "Vascular Disease*" OR "Rheumatoid Arthritis*" OR RA OR "Autoimmune Disease*" OR "Cystic Fibrosis*" OR Mucoviscidosis OR "Neuromuscular Disease*" OR "Movement Disorder*" OR "Dyskinesia Syndrome*" ) OR AB ( "pre frail*" OR "frail elderly*" OR frail* OR "older adult*" OR stroke* OR "cerebrovascular accident*" OR CVA OR CVAs OR Osteoarthriti* OR Osteoarthro* OR "Degenerative Arthriti*" OR Arthrosis OR "Arthroses" OR "osteoarthrosis deformans" OR "Parkinson's Disease*" OR "Parkinson Disease*" OR "Primary Parkinsonism" OR "Paralysis Agitans" OR "lewy bod*" OR "Lewy Neurites" OR "Type 2 Diabetes" OR "Maturity Onset Diabetes*" OR "Type 2 Diabetes Mellitus" OR MODY OR "Diabetes Mellitus*" OR T2DM OR "Chronic Disease*" OR "Chronic Illness*" OR "chronically ill*" OR "Multiple Sclerosis*" OR "Disseminated sclerosis*" OR "heart fail*" OR "Cardiac Failure" OR "Heart Decompensation" OR "Myocardial Failure" OR "Congestive Heart Failure" OR "Cardiovascular Disease*" OR "Vascular Disease*" OR "Rheumatoid Arthritis*" OR RA OR "Autoimmune Disease*" OR "Cystic Fibrosis*" OR Mucoviscidosis OR "Neuromuscular Disease*" OR "Movement Disorder*" OR "Dyskinesia Syndrome*" ) ) OR ( TI ( ((MH "Walking+") OR ambulat* OR walk* OR movement*) AND difficulty ) OR AB ( ((MH "Walking+") OR ambulat* OR walk* OR movement*) AND difficulty ) ) OR ( (MH "Community Living+") OR (MH "Assisted Living") OR (MH "Disability Evaluation+") OR (MH "Arthroplasty+") ) OR ( TI ( "Mobility Limitation*"OR "Independent Living" OR "community dwelling" OR "Disability Evaluation" OR "Arthroplasty" ) OR AB ( "Mobility Limitation*"OR "Independent Living" OR "community dwelling" OR "Disability Evaluation" OR "Arthroplasty" ) ) ) AND ( ( (MH "Therapeutic Exercise+") OR (MH "Resistance Training") OR (MH "Muscle Strengthening+") OR (MH "Muscle Strength+") OR (MH "Muscles+") OR (MH "Dynamometry") OR (MH "Physical Fitness+") OR (MH "Physical Endurance+") ) OR ( TI ( "exercise therap*" OR "remedial exercis*" OR "rehabilitation exercis*" OR "resistance training" OR "strength training" OR "muscle strength*" OR "Arthrogenic Muscle Inhibition*" OR "Muscle Strength Dynamometer*" OR dynamometer* OR "Physical Fitness*" OR "Physical Endurance*" OR "multicomponent exercise*" ) OR AB ( "exercise therap*" OR "remedial exercis*" OR "rehabilitation exercis*" OR "resistance training" OR "strength training" OR "muscle strength*" OR "Arthrogenic Muscle Inhibition*" OR "Muscle Strength Dynamometer*" OR dynamometer* OR "Physical Fitness*" OR "Physical Endurance*" OR "multicomponent exercise*" ) ) OR ( TI ( (weight) AND (lift* OR bearing) AND (strength* OR exercis* OR program*) ) OR AB ( (weight) AND (lift* OR bearing) AND (strength* OR exercis* OR program*) ) ) ) AND ( (MH "Muscle Fatigue") OR ( TI ( "muscle power*" OR "muscle quality" OR "power output*" OR "high velocity*" AND "low load*" OR "LLHV" OR "power training" OR "functional performance" OR "rate of force*" OR "rate of velocity*" OR "training load" OR (high AND low AND "external resistance") OR "muscle volume*" OR "force velocity*" OR (muscl* AND fatigue) OR "Muscle hypertrophy" OR "Functional outcome*" OR "Muscle mass" OR "peak power" ) OR AB ( "muscle power*" OR "muscle quality" OR "power output*" OR "high velocity*" AND "low load*" OR "LLHV" OR "power training" OR "functional performance" OR "rate of force*" OR "rate of velocity*" OR "training load" OR (high AND low AND "external resistance") OR "muscle volume*" OR "force velocity*" OR (muscl* AND fatigue) OR "Muscle hypertrophy" OR "Functional outcome*" OR "Muscle mass" OR "peak power" ) ) ) | CINAHL | 855 |
| (TS=("pre frail*" OR "frail elderly*" OR frail* OR "older adult*" OR stroke* OR "cerebrovascular accident*" OR CVA OR CVAs OR Osteoarthriti* OR Osteoarthro* OR "Degenerative Arthriti*" OR Arthrosis OR "Arthroses" OR "osteoarthrosis deformans" OR "Parkinson's Disease*" OR "Parkinson Disease*" OR "Primary Parkinsonism" OR "Paralysis Agitans" OR "lewy bod*" OR "Lewy Neurites" OR "Type 2 Diabetes" OR "Maturity Onset Diabetes*" OR "Type 2 Diabetes Mellitus" OR MODY OR "Diabetes Mellitus*" OR T2DM OR "Chronic Disease*" OR "Chronic Illness*" OR "chronically ill*" OR "Multiple Sclerosis*" OR "Disseminated sclerosis*" OR "heart fail*" OR "Cardiac Failure" OR "Heart Decompensation" OR "Myocardial Failure" OR "Congestive Heart Failure" OR "Cardiovascular Disease*" OR "Vascular Disease*" OR "Rheumatoid Arthritis*" OR RA OR "Autoimmune Disease*" OR "Cystic Fibrosis*" OR Mucoviscidosis OR "Neuromuscular Disease*" OR "Movement Disorder*" OR "Dyskinesia Syndrome*") OR TS=((ambulat* OR walk* OR movement*) AND difficulty) OR TS=("Mobility Limitation*" OR "Independent Living" OR "community dwelling" OR "Disability Evaluation" OR "Arthroplasty" )) AND (TS=("exercise therap*" OR "remedial exercis*" OR "rehabilitation exercis*" OR "resistance training" OR "strength training" OR "muscle strength*" OR "Arthrogenic Muscle Inhibition*" OR "Muscle Strength Dynamometer*" OR dynamometer* OR "Physical Fitness*" OR "Physical Endurance*" OR "multicomponent exercise*" OR "therapeutic exercis*" OR Dynamometry) OR TS=((weight) AND (lift* OR bearing) AND (strength* OR exercis* OR program*) ) ) AND (TS=("muscle power*" OR "muscle quality" OR "power output*" OR "high velocity*" AND "low load*" OR "LLHV" OR "power training" OR "functional performance" OR "rate of force*" OR "rate of velocity*" OR "training load" OR (high AND low AND "external resistance") OR "muscle volume*" OR "force velocity*" OR (muscl* AND fatigue) OR "Muscle hypertrophy" OR "Functional outcome*" OR "Muscle mass" OR "peak power" )) | Web of Science | 199 |
| \| frail "muscle power" \| 10 \| These were combined using AND \| \| --- \| --- \| --- \| \| "low load" "high velocity" \| 7 \| These were combined using AND \| \| stroke "muscle power" \| 10 \| These were combined using AND \| \| Osteoarthriti* "muscle power" \| 15 \| These were combined using AND \| \| Arthrosis* "muscle power" \| 0 \| These were combined using AND \| \| Arthriti* "muscle power" \| 2 \| These were combined using AND \| \| "Parkinson's Disease" "muscle power" \| 3 \| These were combined using AND \| \| Parkinson Disease "muscle power" \| 0 \| These were combined using AND \| \| diabetes "muscle power" \| 1 \| These were combined using AND \| \| chronic illness "muscle power" \| 0 \| These were combined using AND \| \| chronic disease "muscle power" \| 1 \| These were combined using AND \| \| multiple sclerosis "muscle power" \| 6 \| These were combined using AND \| \| "autoimmune disease" "muscle power" \| 0 \| These were combined using AND \| \| cystic fibrosis "muscle power" \| 1 \| These were combined using AND \| \| neuromuscular "muscle power" \| 12 \| These were combined using AND \| \| movement disorder "muscle power" \| 0 \| These were combined using AND \| \| lewy body "muscle power" \| 0 \| These were combined using AND \| \| LLHV \| 0 \|  \| \| low load high velocity \| 2 \|  \| \| "rate of force" \| 43 \|  \| \| rate of velocity \| 0 \|  \| \| **TOTAL before deduplication** \| **113** \|  \| \| **After deduplication** \| **104** \|  \| | PEDro | 104 |

**Supplemental Table 2: Cochrane Risk-of-Bias**

|  | Sequence Generation | Allocation concealment | Blinding (personnel) | Blinding (outcome assessment) | Incomplete outcome data | Free selective outcome reporting | Free from other sources of bias |
| --- | --- | --- | --- | --- | --- | --- | --- |
| Sayers 2003 | **-** | **-** | **-** | **-** | **+** | **+** | **+** |
| Reid 2008 | **-** | **-** | **-** | **-** | **+** | **+** | **+** |
| Bean 2009 | **+** | **+** | **-** | **+** | **+** | **+** | **+** |
| Webber 2010 | **+** | **+** | **+** | **+** | **+** | **+** | **+** |
| Zech 2012 | **+** | **+** | **-** | **+** | **+** | **+** | **+** |
| Cadore 2014 | **+** | **+** | **-** | **+** | **+** | **+** | **+** |
| Paul 2014 | **+** | **+** | **-** | **+** | **+** | **+** | **+** |
| Jin 2015 | **-** | **-** | **-** | **-** | **-** | **-** | **-** |
| Kelly 2016 | **+** | **+** | **+** | **+** | **+** | **+** | **+** |
| Medina-Perez 2016 | **+** | **+** | **-** | **+** | **+** | **+** | **+** |
| Ni 2016 | **+** | **+** | **-** | **-** | **+** | **+** | **+** |
| Yoon 2017 | **-** | **-** | **-** | **-** | **+** | **+** | **+** |
| Celes 2017 | **-** | **-** | **-** | **-** | **+** | **+** | **+** |
| Cherup 2019 | **+** | **+** | **+** | **+** | **-** | **+** | **+** |

(+) low risk of bias, (-) unclear, not reported, or high risk of bias
